# Management of a rare case of arrhythmogenic right ventricular dysplasia in pregnancy: a case report

**DOI:** 10.1186/1752-1947-5-300

**Published:** 2011-07-10

**Authors:** Nilgün Güdücü, Salih Serdar Kutay, Ebru Özenç, Çavlan Çiftçi, Alin Başgül Yiğiter, Herman İşçi

**Affiliations:** 1 İstanbul Bilim University, School of Medicine, Department of Obstetrics and Gynecology, Kısıklı cad. No. 106, 34692, İstanbul, Turkey; 2 Ümraniye Education and Research Hospital, Department of Cardiovascular Surgery, İstanbul, Turkey; 3 İstanbul Bilim University, School of Medicine, Department of Cardiology, İstanbul, Turkey

## Abstract

**Introduction:**

Arrhythmogenic right ventricular dysplasia is a heritable disease of the heart muscle characterized by fibrofatty degeneration of cardiomyocytes. Patients present with ventricular arrhythmias or congestive heart failure, and sometimes sudden cardiac death occurs. Prenatal diagnosis has become possible with the detection of mutations, but, to the best of our knowledge, no case of prenatal diagnosis has been reported previously. There is little information about the management of arrhythmogenic right ventricular dysplasia in pregnancy, and the preferred mode of delivery is not certain; therefore, we present the case of a patient with arrhythmogenic right ventricular dysplasia and discuss the prenatal diagnosis, patient management and prognosis in pregnancy.

**Case presentation:**

A 26-year-old Caucasian woman who presented to our hospital with heart palpitations was diagnosed with arrhythmogenic right ventricular dysplasia, and, after three years of follow up with anti-arrhythmic drugs, she wanted to conceive. During pregnancy, she ceased taking her medication. She tolerated pregnancy very well but her cardiac symptoms recurred after her 30th week of pregnancy. She delivered a baby via cesarean section under general anesthesia in her 38th week of pregnancy. She was discharged without any medications and continued lactation for six months.

**Conclusion:**

Patients with mild to moderate arrhythmogenic right ventricular dysplasia tolerate pregnancy and breastfeeding very well, but patients with end-stage arrhythmogenic right ventricular dysplasia should be discouraged from conception.

## Introduction

Arrhythmogenic right ventricular dysplasia (ARVD) is an autosomal dominant inherited disease of the heart muscle characterized by fibrofatty degeneration of cardiomyocytes, which leads to electrical instability and contractility abnormalities. Most patients present with ventricular arrhythmias and later develop right ventricular failure due to progressive muscle damage, but in 7% to 29% of patients the first manifestation of the disease can be sudden cardiac death [1].

Several genes encoding desmosomal proteins have been associated with ARVD [2], and although they can be detected in only 50% of patients, prenatal diagnosis can be considered. Pregnancies with dilated and hypertrophic cardiomyopathies are common, but only a few cases of pregnancies with ARVD have been reported. Therefore, it is difficult to assess the risks of pregnancy and delivery in patients with ARVD. Here we discuss the management of a patient with ARVD during pregnancy, delivery and the postpartum period, together with the possibility of prenatal diagnosis.

## Case presentation

A 26-year-old Caucasian woman presented to cardiology polyclinics with heart palpitations and shortness of breath. The patient's mother had died when she was 35 years old as a result of sudden cardiac arrest (SCA), and her grandmother had died as a result of congestive heart failure (CHF). The patient's body mass index was 24 kg/m^2^. The 24-hour electrocardiographic (ECG) monitoring documented 2602 bi-geminal, tri-geminal and quadri-geminal ventricular extrasystoles per hour as well as ventricular tachycardia (VT) episodes. The duration of filtered QRS was more than 120 ms. Her echocardiogram was within the normal ranges. Cardiac non-contrast-enhanced magnetic resonance imaging (MRI) showed diffuse thinning of the right ventricle and local dilation in the right ventricular wall with segmental hypokinesia. The electrophysiological study revealed sustained non-inducible VT, low-amplitude areas in the right ventricular outflow tract and ventricular ectopic beats originating in the right ventricular outflow tract. Because VT was non-inducible, neither the use of an implanted cardioverter-defibrillator (ICD) nor ablation was considered. There was right ventricular dilation and apical mild hypokinesia. She had no signs of left ventricular dysfunction. On the basis of these results, the diagnosis of ARVD was made according to the original International Task Force diagnostic criteria. She was prescribed metoprolol and propafenone. She avoided physical stress and did very well with pharmacological treatment. After three years of follow-up, she wanted to conceive. She was counseled that only a few pregnancies have been reported in patients with ARVD and that the risk of transmission of the disease to the offspring is 50%. A mutation screening was offered, but she refused the mutation screening because of the high cost. She conceived and ceased her medications but did very well during her pregnancy until term.

A fetal echocardiogram was performed at the 21st week of pregnancy, and after delivery no abnormality was detected. In the third trimester, she had heart palpitations and became symptomatic again. The 24-hour ECG monitoring at the 32nd week of pregnancy documented 16,251 ventricular extrasystoles.

She delivered at the 38th week of pregnancy by elective cesarean section while under general anesthesia. After three days of hospitalization, she was discharged without medications and continued breastfeeding for six months.

## Discussion

The diagnosis of ARVD is based upon a set of major and minor criteria proposed by the International Task Force. Patients must either meet two major criteria comprising one major and two minor criteria or meet four minor criteria to be diagnosed with ARVD. In 1994, the International Task Force proposed criteria for the clinical diagnosis of ARVD [3]. Structural, histological, electrocardiographic, arrhythmic and familial features of the disease were incorporated into the criteria and subdivided into minor and major categories. Although the 1994 criteria were highly specific, they lacked sensitivity for early and familial cases. In 2010, the International Task Force criteria were revised [4]. The revision of the diagnostic criteria provides advances in the genetics of ARVD. To improve sensitivity, quantitative criteria were proposed and abnormalities were compared with the values of healthy individuals. On the basis of this knowledge, in our case the patient's presentation is consistent with the original International Task Force Criteria comprising four minor criteria for the diagnosis. One criterion is hypokinesis of the right ventricle observed by MRI and angiography. However, the MRI, angiographic and echocardiographic findings that we used to diagnosis our patient with ARVD apparently are not consistent with the revised International Task Force criteria, because in the revised criteria right ventricular akinesis, dyskinesia and aneurysms are included. On the other hand, the new arrhythmia, ECG and family history criteria support our diagnosis with four minor criteria. ARVD has variable manifestations, and disease progression occurs in several phases; therefore, our patient will continue to be followed up closely in the coming years.

ARVD has variable penetrance and incomplete expression [5]. The inheritance of a mutation previously identified in a proband does not imply a diagnosis of ARVD when clinical diagnostic criteria are not met. Genetic analysis cannot predict clinical phenotype and risk stratification, but can enable exclusion from lifelong clinical follow-up when a mutation is not detectable. Seven mutations at different loci have been associated with ARVD, but they do not explain intra-familial variation and gender differences. Our patient refused mutation screening because of the high cost. Although genetic screening and prenatal diagnosis are possible, a prenatally diagnosed mutation has not been reported yet. When a mutation is detected prenatally, this does not mean that the offspring will certainly develop ARVD phenotype in the future, so it is not ethical to terminate the pregnancy even if the mutation is present. The age of onset of symptoms has been reported to be earlier in the younger generations compared to older generations [6]. The family history in our case supports this theory because the disease appeared at an earlier age in the coming generation and decreased life expectancy. If these characteristics are supported in future case reports, genetic screening and prenatal diagnosis may become common.

Mutations affecting components of the cardiac desmosomes are believed to disrupt cell-to-cell adhesion, leading to the detachment of myocytes under conditions of mechanical stress [7]. This in turn might lead to apoptosis and cell necrosis and then to inflammation and repair by fibrofatty tissue.

During pregnancy, plasma volume, cardiac output and heart rate increase, hematocrit decreases and physiologic anemia are established. These changes are required for the adaptation of the cardiovascular system to pregnancy. In the presence of maternal heart disease, the patient's hematocrit level should be kept between 30% and 35% to prevent complications. Only a few pregnancies associated with ARVD have been reported, and, to the best of our knowledge, there has been no reported pregnancy associated with end-stage ARVD. Therefore, it is not possible to predict whether patients with ARVD are able to tolerate the hemodynamic changes of pregnancy. Our patient tolerated pregnancy very well, and no progression of disease extent was detected on the basis of 24-hour ECG and echocardiography at the 32nd week of pregnancy and after delivery. As reported previously, patients with a mild or moderate form of the disease tolerate the hemodynamic changes due to pregnancy well, but some of them become symptomatic in the last trimester and puerperium [8]. Our patient refused to take her medications during pregnancy and she fared very well until full term. She experienced some heart palpitations and chest discomfort after the 32nd week of pregnancy. She was advised to take β-blockers, as these drugs are considered relatively safe in pregnancy, but she refused to take these drugs. Treatment of ARVD is based on the prevention of arrhythmia with β-blockers, especially with sotalol, which prevents arrhythmia caused by ARVD in 60% to 70% of patients [9]. Propafenone is reported to be safe in the treatment of ventricular arrhythmia during pregnancy [10]. ICD use is recommended for patients who have had a documented episode of sustained VT or cardiac arrest or who have high-risk features for SCA [11]. ICD can prevent sudden cardiac death in early- to mid-stage ARVD, but in end-stage ARVD with CHF, the prognosis is poor [12]. Patients with end-stage ARVD and CHF should avoid pregnancy.

In the puerperial period, our patient refused starting anti-arrhythmic drug therapy because she wanted to breastfeed. Although lactation has been reported to decrease maternal electrolyte stores [13] and is not advised, our patient's weekly magnesium, sodium and potassium plasma levels were normal during the puerperial period. Therefore, we do not advise abstaining from breastfeeding in early- or mid-stage ARVD, when the patient is not using drugs or when the drugs are safe for breastfeeding.

Ten percent of sudden deaths of patients with ARVD occur in stressful conditions and 10% occur in the peri-operative period [14]. Only a few cases of pregnancy in patients with ARVD have been reported. Although the preferred mode of delivery in women with ARVD is not certain, Bauce *et al*. [8] reported six cases of pregnancies in women with ARVD. Four of these women chose delivery by cesarean section, and two patients with less severe ARVD allowed vaginal delivery. We preferred to perform elective cesarean section in our patient while she was under general anesthesia to avoid strenuous labor and the hemodynamic changes of epidural anesthesia. Cardiovascular collapse during anesthesia in patients with ARVD has been reported to be unresponsive to resuscitation, and most of these patients did not have pre-existent arrhythmia [15].

## Conclusion

Pregnancies complicated by mild to moderate ARVD can be managed successfully with close monitoring and anti-arrhythmic drugs when necessary, but patients with end-stage ARVD should be discouraged from conceiving. Breastfeeding should be permitted in patients with mild to moderate ARVD with close electrolyte monitoring. Prenatal screening is possible, but terminating the pregnancy after the detection of a mutation does not seem ethical.

## Consent

Written informed consent was obtained from the patient for publication of this case report and any accompanying images. A copy of the written consent is available for review by the Editor-in-Chief of this journal.

## Competing interests

The authors declare that they have no competing interests.

## Authors' contributions

NG and ÇÇ conducted patient follow-up during the patient's pregnancy in the obstetrics and cardiology polyclinics. SSK interpreted the patient data. Hİ and EÖ were the attending surgeons for the cesarean section. ABY provided the peri-natology consultation. NG was the major contributor to the manuscript. All authors read and approved the final manuscript.
